# The Role of AlphαSynuclein in Mouse Models of Acute, Inflammatory and Neuropathic Pain

**DOI:** 10.3390/cells11121967

**Published:** 2022-06-19

**Authors:** Moritz Möller, Christine V. Möser, Ulrike Weiß, Ellen Niederberger

**Affiliations:** 1 Pharmazentrum Frankfurt/ZAFES, Institut für Klinische Pharmakologie, Klinikum der Goethe-Universität Frankfurt, Theodor Stern Kai 7, 60590 Frankfurt am Main, Germany; m_moeller@mail.de (M.M.); chmoeser@hotmail.com (C.V.M.); weiss-ulrike@gmx.de (U.W.); 2 Fraunhofer Institute for Translational Medicine and Pharmacology ITMP, Theodor Stern-Kai 7, 60590 Frankfurt am Main, Germany

**Keywords:** alphaSynuclein, nociception, inflammation, neuropathic pain, MAP kinase

## Abstract

(1) AlphαSynuclein (αSyn) is a synaptic protein which is expressed in the nervous system and has been linked to neurodegenerative diseases, in particular Parkinson’s disease (PD). Symptoms of PD are mainly due to overexpression and aggregation of αSyn and include pain. However, the interconnection of αSyn and pain has not been clarified so far. (2) We investigated the potential effects of a αSyn knock-out on the nociceptive behaviour in mouse models of acute, inflammatory and neuropathic pain. Furthermore, we assessed the impact of αSyn deletion on pain-related cellular and molecular mechanisms in the spinal cord in these models. (3) Our results showed a reduction of acute cold nociception in αSyn knock-out mice while responses to acute heat and mechanical noxious stimulation were similar in wild type and knock-out mice. Inflammatory nociception was not affected by αSyn knock-out which is also mirrored by unaltered inflammatory gene expression. In contrast, in the SNI model of neuropathic pain, αSyn knock-out mice showed decreased mechanical allodynia as compared to wild type mice. This effect was associated with reduced proinflammatory mechanisms and suppressed activation of MAP kinase signalling in the spinal cord while endogenous antinociceptive mechanisms are not inhibited. (4) Our data indicate that αSyn plays a role in neuropathy and its inhibition might be useful to ameliorate pain symptoms after nerve injury.

## 1. Introduction

α-synuclein (αSyn) is a small soluble protein of 14 kD that is expressed in the central and the peripheral nervous system, enriched in neurons and mainly located in synaptic vesicles of presynaptic nerve terminals [1,2]. Although the protein has been known for a long time, its physiological functions are not fully understood. It is suggested to be involved in regulatory processes [3] by its function in exocytotic processes [2,4] as well as the recycling of synaptic vesicles [5], thereby playing a role in synaptic transmission and plasticity [6,7]. αSyn is primarily known as a contributor in the pathogenesis of Parkinson’s disease (PD), in which mutations, polymorphisms and multiplications of the protein have been associated with sporadic and familial forms of the disease [8,9,10]. In particular, the overexpression and aggregation of α-Syn contributes to a degradation of dopaminergic neurons [11,12], leading to the typical PD-associated motor disturbances such as tremor and rigidity. Other, non-motor symptoms comprise olfactory disturbances such as dystonia, but also psychic diseases such as dementia, anxiety and depression [13,14,15]. Furthermore, about 60–70% of PD patients experience chronic pain, often in the prodromal phase of PD long before the manifestation of motor problems but also in advanced stages. The patients suffer from different types of pain including acute or chronic forms of e.g., musculoskeletal, nocturnal, orofacial, central and peripheral pain [16,17,18,19]. The genesis of these types of pain is multifactorial and suggested to be at least partially based on mechanisms in the CNS [20]; however, the origins and mechanisms of pain in PD have not been completely clarified as of yet. It has been suggested that a dysfunctional pain processing in the striatum [21], insufficient pain inhibition and central sensitization are reasons for the development of PD-associated pain, which could be supported by functional imaging showing pathological activation areas in PD patients after nociceptive stimulation [22]. The occurrence of pain appears independent of degradation of dopaminergic neurons, since dopamine therapy has no effect on pain symptoms in PD patients [23,24]. Although several models of parkinsonism have been established in rodents, the mechanisms contributing to nociception have been mostly neglected so far. It has already been suggested that the injection of αSyn fibrils in mice contributes to development of pain hypersensitivity [25]. However, overexpression of alphaSynuclein bears the risk of developing peripheral sensory neuropathies with sensory losses which do not allow accurate investigation of pain behavior which is mostly dependent on proper perception of the stimulus and sensorimotor coupling. Therefore, investigating the role of synuclein in synuclein knock-out mice might be a more promising approach to gain insights into its role in nociception. In principle, pain can be roughly distinguished into physiological, pathophysiological and neuropathic pain, the latter two bearing the risk of chronification. Acute pain is an important body protection system that prevents severe harm. In contrast, chronic pain is either associated with increased excitatory nociceptive transmission or with a decrease in endogenous central pain inhibition. The mechanisms involve activation of inflammatory cells and genes as well as signal transduction pathways activating kinases and transcription factors, for example, thereby leading to the transcription of further pain genes. Endogenous antinociceptive pathways including endogenous opioids or inhibitory neurotransmitters are disturbed. Ongoing inflammation or tissue/nerve damage can induce these molecular changes in the central and peripheral nervous system, leading to pain hypersensitivity, referred to as central and peripheral sensitization [26,27]. The aim of this study was to clarify possible functions of αSyn in acute, inflammatory, and neuropathic pain. Therefore, we applied a Snca knock-out mouse model and performed behavioral experiments to assess nociceptive responses after stimulation with acute thermal and mechanical noxious stimuli. Furthermore, we induced inflammatory and neuropathic pain and measured spontaneous pain-like behavior or pain hypersensitivity, respectively. In addition, we used RT-PCR, western blot and immunofluorescence analysis to analyze the cellular and molecular mechanisms underlying potential differences between wild type and Snca knock-out mice in pain-relevant tissues. Our results indicate a role of αSyn mainly in the regulation of neuropathic pain through the downregulation of the inflammatory pathway and MAP Kinase signaling in the spinal cord.

## 2. Materials and Methods

### 2.1. Animals

Male C57BL/6 mice were obtained from Charles River, Sulzfeld, Germany at the age of six to eight weeks. Homozygous B6; 129 × 1-Sncatm1Rosl/J^−/−^ mice with a mixed C57BL/6; 129 background were purchased from Jackson Laboratories. In these mice, the exons 1 and 2 of the Snca gene encoding amino acids 1–41 were disrupted. Snca^−/−^ mice are viable, fertile and healthy [28]. It has been described that knock-out animals have a reduced number of synaptic vesicles in hippocampal neurons [28] and may show increased anxiety in comparison with wild type animals. However, motor functions and learning ability do not differ from wild types [29]. Snca^−/−^ mice were backcrossed with C57BL/6 wild type mice to obtain heterozygous offspring which were then mated to receive wild type and Snca^−/−^ littermates. Genotyping was performed using the following primers as recommended by the supplier:
194035′- TCA GCC ACG ATA AAA CTG AGG -3′194045′- GTG AGG GCT GTG GGT ATC TG -3′oIMR84445′- GCC TGA AGA ACG AGA TCA GC -3′

Animals had free access to food and water and were maintained in climate- and light-controlled rooms (24 ± 0.5 °C, 12/12 dark/light cycle). In all experiments, the European ethical guidelines for investigations in conscious animals were obeyed and the procedures were approved by the local Ethics Committee for Animal Research (Regierungspräsidium Darmstadt, Approval Number FK1081). All efforts were made to minimize animal suffering and to reduce the number of animals used. All behavioral experiments were performed by an observer blinded to the genotype in a dedicated room with restrictions on sound level and activity.

### 2.2. Nerve Injury

The spared nerve injury (SNI) model was used as described previously [30,31]. Briefly, animals were anesthetized with isoflurane, and the tibial and common peroneal branches of the sciatic nerve were ligated and sectioned distally, whereas the sural nerve was left intact. For sham surgery, the sciatic nerve was exposed but not touched. Sham operated mice were used as controls. Surgical pain was reduced by topical administration of lidocaine. Mechanical sensitivity was assessed at baseline, 7 d, 14 d, 21 d and 28 d after surgery. Animals were sacrificed at the indicated time points after surgery and the lumbar spinal cord (L4–5) was dissected for further analysis.

### 2.3. Behavioral Testing

Snca^−/−^- and Snca^+/+^-littermates were used in all behavioural tests. Before the start of the experiments, mice were placed into the respective experimental setting in the experimental room for at least 30 min for habituation. Experiments were performed by the same observer blinded to the genotype.

#### 2.3.1. Rotarod Test

A Rota Rod treadmill for mice (Ugo Basile, Comerio, Italy) at a constant rotating speed of 32 rpm was used to assess motor coordination. All mice had five training sessions prior to the day of the experiment. The latency until mice fell from the rotating rod was analysed with an upper cut-off time of 90 s. The fall-off latency was averaged from five tests.

#### 2.3.2. Mechanical Sensitivity

Mechanical sensitivity was assessed by an automated testing device (Dynamic Plantar Aesthesiometer, Ugo Basile, Varese, Italy) consisting of a steel rod which is pushed against the plantar surface of the paw with increasing force until the paw is withdrawn (paw withdrawal latency, PWL). The maximum force was set at 5 g to prevent tissue damage and the ramp speed was 0.5 g/s (Cut-off 20 s). Mice were allowed to habituate to test cages with a metal grid bottom for 1 h. The paw withdrawal latencies of both paws were then obtained as mean of four to six consecutive trials at each time point (at least 30 s between repeated measurements of the same paw). For analysis of mechanical allodynia, a ratio for the paw withdrawal latency between ipsi- and the contralateral paw was calculated as (ipsi-contra)/contra*100.

#### 2.3.3. Hot/Cold Plate Test

The device was either heated up to 52 °C or cooled down to 4 °C. The animal was then placed on the metal plate of the Hot/Cold Plate test. The time was stopped until the animal showed some avoidance or painful behaviour, e.g., walking up the plastic cage, licking/flinching of the paws, or jumping. The cut off time to avoid tissue damage was 25 s for the hot plate test, and 40 s for the cold plate test. Each animal was tested only once, since repeated testing in this assay can lead to latency changes [32].

#### 2.3.4. Formalin Test

The formalin test was performed as described [33]. Briefly, mice were placed in a plexiglass rectangle and were allowed to acclimatize for 30 min. Twenty µL of a 5% formalin solution was injected subcutaneously into the dorsal surface of the left hind paw. Directly after formalin injection, the time mice spent licking the formalin-injected paw was assessed in 5-min intervals up to 45 min.

#### 2.3.5. Zymosan-Induced Paw Inflammation

After assessing mechanical baseline paw withdrawal latencies using the Dynamic Plantar Aesthesiometer as described above, hind paw inflammation was induced by subcutaneous injection of 20 µL of a 10 mg/mL zymosan A (Sigma-Aldrich, Munich, Germany) suspension in phosphate-buffered saline (0.1 M, pH 7.4) into the mid plantar region of the left hind paw [34]. Four consecutive measurements were performed in 10-s intervals hourly up to 8 h and at 24 and 48 h after zymosan injection.

#### 2.3.6. Cold Allodynia

Cold allodynia was assessed in the SNI-model by applying a drop of acetone onto the plantar side of the hindpaw ipsilateral to the nerve lesion acetone with help of a 1 mL syringe. The time the mice spent lifting, shaking or licking the acetone treated paw was recorded during an observation period of 2 min starting right after acetone application.

### 2.4. Western Blot Analysis

For Western Blot analysis, mice were treated as indicated. At the indicated time points, mice were killed by CO_2_ and cardiac puncture and the lumbar spinal cords (L4/L5) and the DRGs (L4/L5) were dissected and immediately frozen in liquid nitrogen. Tissues were homogenized in a PhosphoSafe Extraction Buffer (Merck, Darmstadt, Germany) containing protease inhibitor (1 mM Pefabloc SC, Alexis Biochemicals, Lausen, Switzerland). Subsequently, extracts were centrifuged at 14.000 rpm for 1 h at 4 °C to remove cellular debris. The supernatants were stored at −80 °C.

Proteins (30 µg) were separated electrophoretically by 12% SDS-PAGE and then transferred onto nitrocellulose membranes by wet-blotting at 50 V for 90 min (Bio-Rad, München, Germany). To confirm equal loading, all blots were stained with Ponceau red solution. Thereafter, proteins were fixated onto the membrane by 30 min treatment with a 0.4% paraformaldehyde solution. Membranes were blocked for 60 min at room temperature in Odyssey blocking reagent (LI-COR Biosciences, Bad Homburg, Germany) diluted 1:2 in 0.1 M phosphate-buffered saline (PBS), pH 7.4. The blots were then incubated overnight at 4 °C with primary antibodies against alphαSynuclein (BD Bioscience, Franklin Lanes, Evansville, IN, USA, 1:500), p-p38, p-38, p-p42/44, p42/44, (all Cell Signalling Technology, Boston, MA, USA, 1:250), COX-2 (Santa Cruz Biotechnology, Heidelberg, Germany, 1:500), CB1R, (Cayman, Biomol, Hamburg, Germany 1:500), DOR (Abcam, Cambridge, UK, 1:200), Pgp9.5 (Abcam, Cambridge, UK, 1:200) and TRPM8 (alamone labs, Jerusalem, Israel, 1:100) in blocking buffer. After washing three times with 0.1% Tween 20 in PBS, the Blots were incubated for 60 min with an IRDye 800- or IRDye 700-conjugated secondary antibody (Molecular Probes, 1:10.000 in blocking buffer). After rinsing three times in 0.1% Tween 20 in PBS, protein-antibody complexes were detected with the Odyssey Infrared Imaging System (LI-COR, Bad Homburg, Germany). β-actin (1:1.200, Sigma, Munich, Germany) were used as loading controls. Densitometric analysis of the blots was performed with Image Studio Lite software (LI-COR, Bad Homburg, Germany).

### 2.5. Immunofluorescence

Mice were perfused intracardially with 0.1 M phosphate-buffered saline followed by 2% paraformaldehyde (PFA) in 0.1 M PBS, pH 7.4, under deep CO_2_-induced anesthesia. Spinal cords (L4/L5) were dissected, post-fixed in 2% PFA in 0.1 M PBS (pH 7.4), cryoprotected in 20% sucrose in 0.1 M PBS overnight at 4 °C and then embedded in Tissue-Tek O.C.T. Compound (Sakura Finetek Europe B.V., Alphen aan den Rijn, Netherlands) frozen on dry ice. Cryostat sections were cut at a thickness of 16 µm and stored at −80 °C. For immunofluorescence, slices were permeabilized for 10 min with PBS containing 0.1% Triton-X 100. The sections were then blocked in 3% BSA/10% NGS in PBS for 1 h to reduce non-specific bindings. After rinsing in PBS, sections were incubated with antibodies against αSyn (BD Biosciences, Franklin Lanes, Evansville, IN, USA, 1:100), CGRP (Santa Cruz Biotechnology, Heidelberg, Germany, 1:200), IB4 (Thermo Fisher Scientific, Langenselbold, Germany, 1:50), GABA-A (Synaptic Systems, Göttingen, Germany, 1:500), GAD67 (Abcam, Cambridge, UK, 1:200), GFAP (Sigma, Deisenhofen, Germany, 1:500), CD11b (WAKO, Neuss, Germany, 1:100). Antibodies were dissolved in PBS/3% BSA and incubated at 4 °C overnight. After rinsing in PBSTx (0.1% Triton), sections were incubated for 2 h at room temperature with Cy3- (Sigma, Deisenhofen, Germany 1:1.000) or Alexa Fluor 488-conjugated secondary antibodies (Molecular Probes, Eugene, OR, USA; 1:1.000) dissolved in PBSTx (0.1% Triton), rinsed again in PBS and coverslipped with Aqua-Poly/Mount (Polysciences, Warrington, Bucks County, PA, USA). Images were captured using an inverted fluorescence microscope (Axio Observer.Z1, Zeiss, Germany) equipped with a monochrome CCD camera and AxioVision software (Zeiss, Germany) or a Keyence BIOREVO microscope. Immunofluorescence images shown in the figures represent only a representative result obtained from at least 3 animals per group. A semiquantitative analysis of the immunofluorescence signals was performed with ImageJ.

### 2.6. Glutamate Measurement in the Cerebrospinal Fluid (CSF)

For the glutamate measurement in cerebrospinal fluid, Promega’s Glutamate-Glo assay was used according to the manufacturer’s protocol. For CSF measurement, CSF and detection reagent were mixed in an equal volume of 5–10 μL, mixed thoroughly for 30–60 s and then incubated for 60 min at RT. Luminescence was measured with a TECAN infiniteF200PRO microplate reader (TECAN, Männedorf, Switzerland).

### 2.7. Real-Time PCR

RNA was prepared from the lumbar spinal cords (L4/L5) using an RNeasy Lipid Tissue Mini Kit (Qiagen, Hilden, Germany) according to the manufacturer’s instructions. Two hundred ng of total RNA was used for the reverse transcription which was performed with Random Primers in a Verso cDNA Kit (ThermoFisher Scientific, Darmstadt, Germany), respectively. Twenty ng of RNA equivalent were subjected to real-time PCR in an Applied Biosystems sequence detection system Quant 5 using a SYBR Select Master Mix Kit (ThermoFisher Scientific, Darmstadt, Germany). The expression of CD11b, COX-2, GFAP, IL-1β and TNFα mRNA was determined and normalized to GAPDH mRNA.

The following gen-specific primers were used:
CD11bFW 5′-CTGCCTCAGGGATCCGGAAAG-3′
RV 5′-TGTCTGCCTCGGGGATGACATC-3′COX-2FW 5′-AGACACTCAGGTAGACATGATCTACCCT-3′
RV 5′-GGCACCAGACCAAAGACTTCC-3′GFAPFW 5′-AGAACAACCTGGCTGCGTAT-3′
RV 5′-TCCTCCTCCAGCGATTCAAC-3′IL1βFW 5′-CTGGTGTGTGACGTTCCCATTA-3′
RV 5′-CCGACAGCACGAGGCTTT-3′TNFαFW 5′-GCTGAGCTCAAACCCTGGTA-3′
RV 5′-CGGACTCCGCAAAGTCTAAG-3′GAPDHFW 5′-CAATGTGTCCGTCGTGGATCT-3′
RV 5′-GTCCTCAGTGTAGCCCAAGATG-3′

The cycle number at which the fluorescence signals cross a defined threshold (Ct-value) is proportional to the number of RNA copies present at the start of the PCR. The threshold cycle number for the specific mRNA was standardized by subtracting the Ct-value of GAPDH from the Ct-value of the specific genes of the same sample, respectively. The relative quantitative level of samples was determined by standard 2^(−ddCt)^ calculations and expressed as fold-change of a reference control sample.

### 2.8. Data Analysis

Statistical evaluation was done with Graph Pad Prism 9.3.1 for Windows. Data are presented as mean ± SEM. Data were compared by one-way or two-way univariate analysis of variance (ANOVA) with subsequent t-tests employing a Bonferroni α-correction for multiple comparisons or Dunnett’s post hoc test, respectively. In cases of only two groups, a Student’s two-tailed, unpaired t-test was used. The number of animals used or repetitions (n) for every experiment are given in the figure legends. For analysis of mechanical allodynia, a ratio for the paw withdrawal latency between ipsi- and the contralateral paw was calculated as (ipsi-contra)/contra*100. In addition, the area under the paw withdrawal latency versus time curve (AUC) was calculated by the linear trapezoidal rule. For all tests, a probability value *p* < 0.05 was considered statistically significant.

## 3. Results

### 3.1. Expression and Localization of αSyn in the Spinal Cord

**Figure 1:**
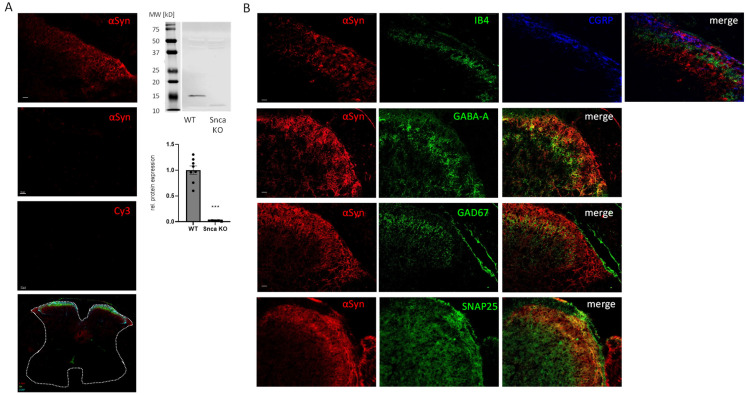
Expression of αSyn in the murine spinal cord. (**A**) left: Immunofluorescence showing (from top to bottom) αSyn expression in the dorsal horn of the spinal cord of wild type and Snca knock-out mice as well as background control stain without primary antibody (Cy3) (Scale bar = 20 µm), an overview on colocalization of αSyn (red), CGRP (turquoise) and IB4 (green) in a complete cross-section of the spinal cord (marked by the dotted line) showing that αSyn is exclusively expressed in the dorsal horn, Scale bar = 200 µm. Right: Western Blot analysis showing expression of αSyn in wild type mice in comparison to Snca knock-out mice. The diagram shows the densitometric analysis of WT vs. Snca knock-out mice (*n* = 8 WT/5 KO), blacks squares and circles show the single measurements (circles = wildtype, squares = Snca knock-out), *** p > 0.001 statistically significant difference between wild type and Snca knock-out mice. (**B**) Upper lane: Colocalization of αSyn (red), CGRP (blue) and IB4 (green) in the dorsal horn of the spinal cord of wild type mice. Upper middle lane: Colocalization of αSyn (red) and GABA-A (green). Lower middle lane: Colocalization of αSyn (red) and GAD67 (green), Lower lane: Colocalization of αSyn (red) and SNAP25 (green) Scale bar = 20 µm (*n* = 3).

Western Blot and immunofluorescence experiments verified that αSyn is expressed in the spinal cord of Snca wild type but not of knock-out mice. In addition, immunofluorescence confirmed previous results [35] revealing the expression of αSyn mainly in laminae 1 and 2 of the dorsal horn which constitute important pain-relevant regions in the spinal cord where mechanisms of central sensitization take place. αSyn immunofluorescence colocalized with the lamina 2 marker isolectin B4 (IB4) and the lamina I marker calcitonin-gene-related protein (CGRP), indicating that α-Syn is expressed in peptidergic and non-peptidergic nociceptive neurons in the dorsal horn of the spinal cord, thus suggesting a potential role in pain transmission in the CNS. In addition, we could show a colocalization of αSyn with GAD67 and GABA-A as markers for GABAergic inhibitory neurons which might serve as a hint that αSyn is involved in endogenous antinociceptive transmission. To show the vesicular localization of αSyn we further performed costaining with the vesicle protein SNAP25 and detected the colocalization of both proteins (Figure 1). In addition to the spinal cord, we also assessed the localization of αSyn in the DRGs and also observed colocalization with markers for petidergic (CGRP) and non-petidergic (IB4) nociceptive neurons as well as large motor neurons (NF200), indicating a potential role of αSyn in peripheral nociceptive processing but also in motor functions (Supplementary Figure S1A).

### 3.2. Effects of αSyn Knock-Out on Motor Function and Acute Nociception

**Figure 2:**
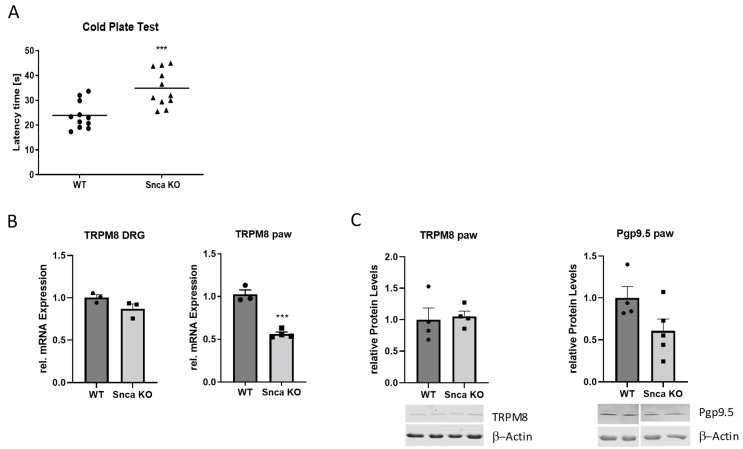
Acute cold nociception and related gene expression. (**A**) nociceptive threshold in the cold plate test. (*n* = 11) (**B**) TRPM8 mRNA expression in the DRGs and the paws of wild type and Snca knock-out mice as assessed by RT-PCR analysis (relative gene expression of TRPM8 in relation to GAPDH). (*n* = 3–4) (**C**) Western blot analysis of Pgp9.5 and TRPM8 in the paws of wild type and Snca knock-out mice; the blots show representative results and the diagrams the densitometric analysis of all samples (*n* = 4). *** *p* < 0.001, statistically significant difference between wild type and Snca knock-out mice. The individual measurements are indicated by the black circles (wild type) and squares (Snca knock-out).

Intact motor function is an important prerequisite to assess nociceptive reactions since these are mostly based on a motor response. We investigated the ability of Snca wild type and knock-out mice in the hanging wire and the rotarod test. The results showed no differences between the respective groups indicating intact motor skills in Snca knock-out mice. In the dynamic plantar test, the Snca knock-out mice showed no signs of mechanical hypersensitivity and, in models for thermal acute nociception, no difference could be observed in the hot plate test. However, cold nociception was significantly suppressed in Snca knock-out in comparison to wild type mice as shown by an increase in latency time until the first reaction (Table 1, Figure 2). To further assess potential mechanisms, which contribute to the reduced acute cold nociception, we analyzed the expression of TRPM8, a cold nociceptor, and Pgp9.5, a marker for peripheral nerve fibers. The results showed a reduced RNA expression of TRPM8 in the paw of Snca knock-out mice which, however, did not occur on the protein level. In the DRGs, there was no regulation of TRPM8 RNA expression. Pgp9.5 protein levels in the paw were slightly but not significantly reduced in Snca knock-out mice (Figure 2).

### 3.3. αSyn Does Not Affect Inflammatory Nociceptive Responses

**Figure 3:**
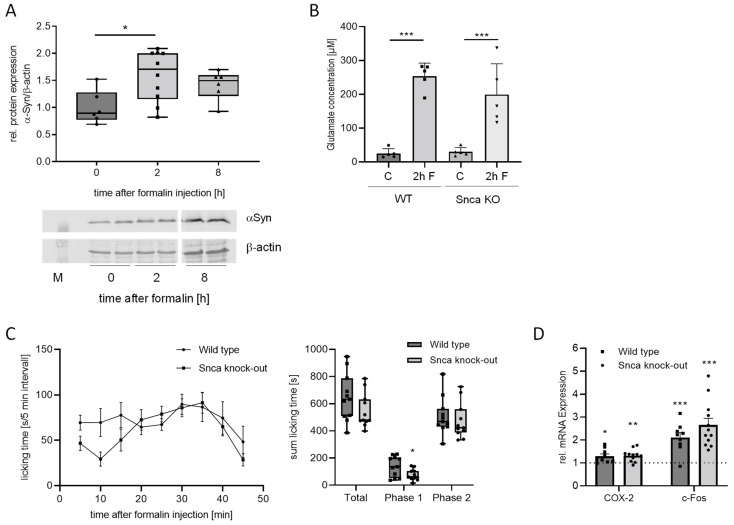
Effects of αSyn deletion in the formalin test. (**A**) Western blot analyses showing αSyn protein expression in the spinal cord 2 and 8 h after formalin injection into the paw of wild type mice. The western blot shows a representative result, the diagram a densitometric analysis of all samples (*n* = 6–8). (**B**) Glutamate concentration in the CSF 2 h after formalin injection in wild type and Snca knock-out mice in comparison to untreated controls (*n* = 5–6), *** *p* < 0.001 indicates a statistically significant difference in comparison with the respective control. (**C**) left panel: time course of the licking behavior of wild type and Snca knock-out mice in 5 min intervals, right panel: sum of licking time over the total observation period of 45 min, in phase 1 (0–10 min) and phase 2 (11–45 min), respectively (*n* = 11). * *p* < 0.05 indicates a statistically significant difference in comparison to wild type phase 1. (**D**) Regulation of representative genes in the spinal cord of wild type and Snca knock-out mice 2 h after formalin injection into the paw. The dotted line indicates the untreated control value, which has been set as 1 (*n* = 8–12), * *p* > 0.05, ** *p* < 0.01, *** *p* < 0.001 indicates a statistically significant difference in comparison to the untreated control. The individual measurements are indicated by the black circles, squares and triangles.

To evaluate a potential role of αSyn in inflammatory nociception, we applied two different mouse models. These are the formalin model, which constitutes a correlate for early central sensitization, and the zymosan-induced paw inflammation model that is suitable to investigate mechanisms of chronic inflammatory pain including cardinal inflammatory symptoms such as edema (swelling) and redness. First, we assessed the regulation of αSyn in the spinal cord after injection of formalin into the hind paw. Two h after formalin administration, we observed a significant upregulation of αSyn protein in the spinal cord, which is still detectable after 8 h (Figure 3A). In the DRGs, we did not detect regulations of αSyn 2 and 8 h after formalin injection (Supplementary Figure S1B). Since αSyn is involved in neurotransmitter regulation, we also investigated the concentration of glutamate, the most important excitatory neurotransmitter in nociception, in the cerebrospinal fluid (CSF) after formalin injection in wild type and Snca^−/−^ mice. A formalin injection into the hind paw induced a significant increase of glutamate in the CSF in both genotypes, although without any difference between Snca wild type and knock-out mice (Figure 3B), which is in accordance with previous publications [36]. The nociceptive behavior of Snca knock-out mice in the inflammatory second phase of the formalin test (11–45 min after formalin injection) was also not altered in comparison to wild type mice. Interestingly, there was a significant reduction in the nociceptive behavior in the first phase (0–10 min) in the knock-out animals (Figure 3C). Similar to the results in the second phase of the formalin test, we did not observe differences in the nociceptive response between wild type and Snca knock-out mice in the zymosan test (Supplementary Figure S2), supporting the assumption that Snca plays only a minor role in inflammatory nociception. This is further strengthened by RT-PCR analyses showing no differences between the two groups concerning the expression of the proinflammatory gene cyclooxygenase 2 (COX-2) as well as the neuronal activity marker c-fos (Figure 3D).

### 3.4. Impact of αSyn on Neuropathic Pain in the SNI Model

**Figure 4:**
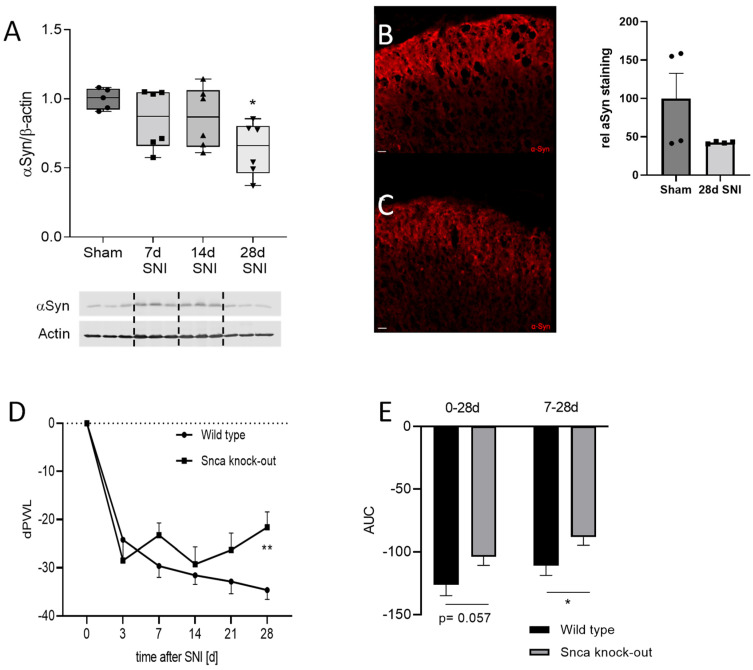
Effects of αSyn in SNI-induced neuropathy (**A**–**C**) Expression of αSyn in the SNI model for neuropathic pain (**A**) western blot analysis showing the time course of αSyn expression over a period of 28 d after SNI in comparison to sham which has been set as 1 for better comparison. The blots show representative results, the diagrams the densitometric analysis of all samples (*n* = 5–6/group). * *p* < 0.05 indicates a statistically significant difference in comparison to sham. (**B**,**C**) Immunofluorescence staining of αSyn in the spinal dorsal horn of sham-treated wild type animals (**B**) or 28 d after SNI (**C**) Scale bar = 20 µm. The insert shows a semi-quantitative analysis of all experiments (*n* = 4/group). (**D**,**E**) Mechanical allodynia of wild type and Snca knock-out mice in the SNI model. (**D**) Time course of the delta paw withdrawal latency (dPWL) after SNI surgery, (**E**) Analysis of the area under the paw withdrawal latency versus time curve (AUC) (*n* = 10–11/group). * *p* > 0.05, ** *p* < 0.01 indicates a statistically significant difference in comparison to the wild type control. Iindividual measurements are indicated by the black circles, squares and triangles.

To induce neuropathy, we applied the spared nerve injury model, in which two of the three branches of the sciatic nerve are dissected leading to a strong reduction of the nociceptive mechanical threshold in the dynamic plantar test. Similar to the formalin test, we analyzed protein expression of αSyn during the time course of SNI and observed a reduced protein level of αSyn in the spinal cord in the western blot as well as immunofluorescence analyses 28 d after SNI (Figure 4A–C). In contrast, no differences in αSyn protein expression were detected in the DRGs (Supplementary Figure S1C). In behavioral experiments, Snca knock-out mice showed a delay in the paw withdrawal latency in comparison to wild type mice, indicating reduced mechanical allodynia. Three days after SNI surgery, both Snca knock-out mice and their wild type littermates revealed a reduction of the mechanical pain threshold in the dynamic plantar test, which is a typical indicator for the induction of neuropathy and associated pain. In wild type animals, this threshold decreased further on day seven and remained stable until the end of the 28-day observation period. In contrast, knock-out mice showed inhibition of the pain response and significantly reduced mechanical allodynia from day seven until the end of the observation period as compared to wild-type mice (Figure 4D,E; Supplementary Figure S3A). In addition to mechanical allodynia, we assessed cold allodynia in the acetone test and found a similar tendency to reduced pain hypersensitivity in Snca knock-out mice, however with high inter-individual variation (Supplementary Figure S3B).

### 3.5. Potential Mechanisms of Reduced Neuropathic Pain in Snca Knock-Out Mice

**Figure 5:**
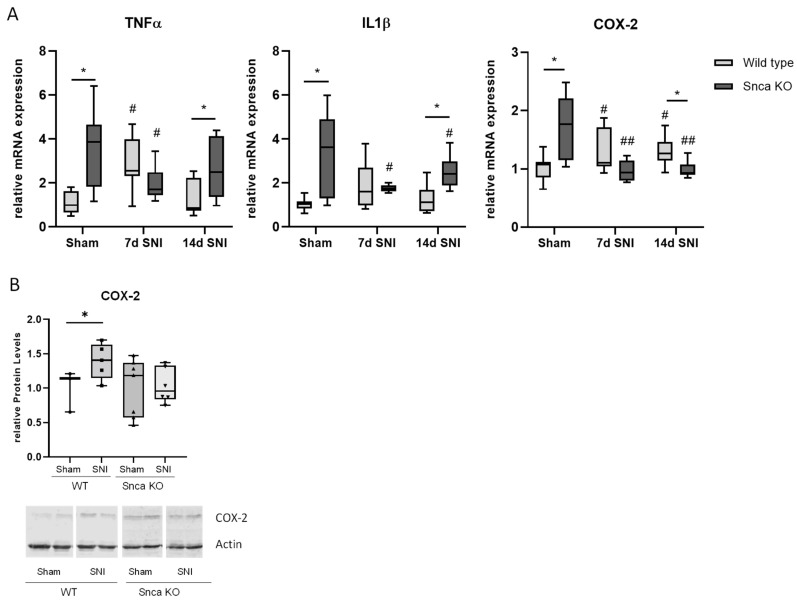

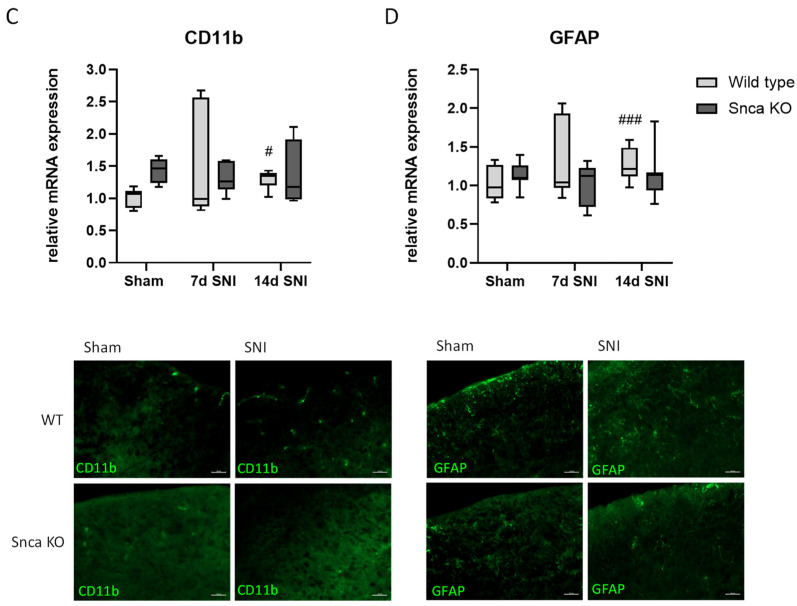
Expression of proinflammatory genes in the spinal cord of mice in the SNI model. (**A**) Relative mRNA expression of IL1β, TNFα and COX-2 in the spinal cord of wild type and SNCA knock-out mice after sham or 7 and 14 d after SNI surgery, respectively. For better comparison, the wild type sham control has been set as 1. (**B**) Western blot analysis showing COX-2 protein levels of Snca wild type and knock-out mice after sham or 14 d SNI treatment, respectively. The blots show representative results, the diagrams the densitometric analysis of all samples. (**C**) RT-PCR and immunofluorescence analysis of the microglia marker CD11b in the spinal cord of sham and SNI treated Snca knock-out mice and SNI treated wild type mice in comparison to the wild type sham control. Scale bars = 20 µm (**D**) RT-PCR and immunofluorescence analysis of the astrocyte marker GFAP in comparison to the wild type sham control. Immunofluorescence showing GFAP staining in the spinal cord of the different treatment groups *n* = 3/group. Scale bars = 20 µm. * p <0.05, statistically significant difference between the genotypes, # *p* < 0.05, ## *p* < 0.01, ### *p* < 0.001 statistically significant difference between Sham and SNI.

To assess potential mechanisms which might contribute to reduced pain-like behavior in the neuropathy model in Snca knock-out mice, we performed RT-PCR, immunofluorescence and western blot analyses with spinal cord tissue. Interestingly, in RT-PCR analyses, a direct comparison of wild type sham controls and Snca knock-out sham controls revealed a significantly increased level of the inflammatory genes COX-2, IL1β and TNFα in the spinal cord of the knock-out mice, which might indicate that Snca knock-out induces overstimulation of the inflammatory transcriptome. After SNI, however, we observed a significant increase of COX-2 and TNFα in the spinal cord of wild type mice 7 and 14 d after SNI surgery in comparison to the wild type sham control, respectively. This increase did not occur in Snca knock-out mice, which showed a significant reduction of COX-2 and TNFα after SNI in comparison to their sham control (Figure 5A). For COX-2 protein, we confirmed the upregulation after SNI in wild type mice, which is completely missing in Snca knock-out mice (Figure 5B). Since glial cells play a major role in neuropathic pain and inflammatory responses, we suspected that invasion and activation of these cells might be involved in the regulation of proinflammatory mediators and therefore performed additional immunostainings and RT-PCR analyses for the microglia marker CD11b and the astrocyte marker GFAP (Figure 5C,D). For CD11b and GFAP, we detected a significant increase in the spinal cord in wild type mice after SNI which was not found in knock-out mice. This result was also observed in immunofluorescence stainings, in which both markers were increased after SNI in wild type mice but not to the same extent in Snca knock-out animals. The occurrence of only small regulations in RT-PCR analyses might be due to the fact that we used complete spinal cord RNA preparations which consist of a mixture of cells and therefore the effects on glial cells might be diluted.

**Figure 6:**
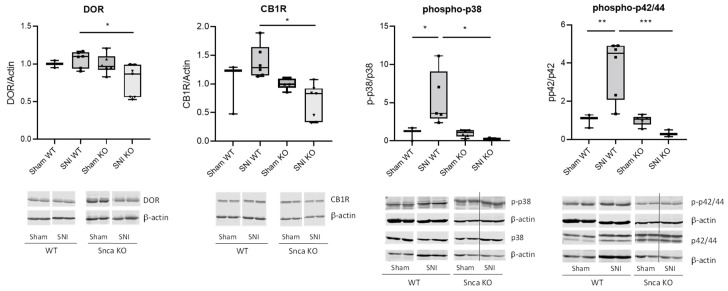
Expression and activity of DOR, CB1R, p38 and p42/44 MAP Kinases in the spinal cords of mice in the SNI model. p-p38 and p-p42/44 are shown in relation to total p38 and p42/44 protein, respectively. All blots were normalized against β-actin. For better comparison, the sham controls were set as 1. The diagrams show the results of all samples, the western blots represent examples from all groups (*n* = 3–6/group) * *p* < 0.05, ** *p* < 0.01, *** *p* < 0.001 indicates a statistically significant difference between the genotypes, (2-way ANOVA).

To address the potential inhibition of antinociceptive pathways, we further investigated the regulation of cannabinoid receptor 1 (CB1) and the delta-opioid receptor (DOR), which have already been associated with αSyn [37,38]. Our results showed no regulation of these genes in wild type mice after SNI on mRNA and protein level. However, and somewhat unexpectedly, in Snca knock-out mice they were significantly downregulated after SNI, indicating that they do not play a role in the reduced nociceptive responses in these mice (Figure 6, Supplementary Figure S4). In the DRGs, we investigated DOR mRNA levels and found no significant regulations in wild type or Snca knock-out mice, respectively (Supplementary Figure S1E). On the other hand, pronociceptive pathways as assessed by MAP Kinase signaling were significantly reduced. Phosphorylation levels of Erk1/2 (p42/44) and p38 MAP kinase in the spinal cord of wild type mice showed the expected significant upregulation of MAPK activity 14 d after SNI; however, this effect is completely lacking in Snca knock-out animals (Figure 6).

## 4. Discussion

Parkinson patients having increased levels of αSyn protein often show pain as an early symptom of the disease. The pathogenesis and the mechanisms of these pain symptoms are not clarified and, to the best of our knowledge, no study has so far dealt with the effects of αSyn downregulation in models of pain. Therefore, in this study, we investigated the impact of αSyn on acute, inflammatory and neuropathic pain in αSyn knock-out mouse models. A potential role of αSyn in nociception is supported by previous publications [35] and in our immunofluorescence data which indicate that αSyn protein is expressed in pain-relevant regions and cells of the spinal cord and the DRGs. Therefore, it might influence nociceptive transmission as well as mechanisms of peripheral and central sensitization. In particular, we showed that αSyn is expressed in glutamatergic excitatory nociceptive neurons as well as GABAergic inhibitory neurons in the dorsal horn of the spinal cord. Since glutamate is the most important excitatory transmitter in pain transmission while GABA constitutes a well-known inhibitory neurotransmitter, the expression of αSyn in these neurons indicates that an αSyn deletion might affect excitatory nociceptive transmission and endogenous central pain inhibition. Dysregulations of these pathways have been associated with chronic pain several times. Previous publications indicate that a deletion of αSyn does not have an effect on excitatory glutamatergic neurotransmitter release [36], which could be supported by our results in the formalin test, in which formalin injection into the paw led to a similar increase of glutamate in the CSF of wild type and Snca knock-out mice. These results suggest that αSyn potentially affects other nociceptive mechanisms or the descending antinociceptive system [39].

Our behavioral results show that a loss of αSyn does not influence acute thermal heat nociception and does not lead to basal mechanical hypersensitivity of Snca knock-out mice. In contrast, it seems to play a role in acute cold nociception which is inhibited in Snca knock-out mice. As potential mechanisms for this effect, we assessed TRPM8 and Pgp9.5 expression in the paw and the DRGs of both genotypes. The only significant effect was the downregulation of TRPM8 RNA in the paws of Snca knock-out mice, which, however, could not be confirmed on the protein level. Pgp9.5 protein in the paw was slightly but not significantly reduced in the knock-out mice. Therefore, we could not identify a distinct mechanism for the ameliorated acute cold pain response of Snca knock-out mice so far.

Results from the formalin test and zymosan-induced paw inflammation indicate that αSyn seems to play only a minor role in inflammatory nociception since no differences could be detected in the reflexive licking behavior in wild type and Snca knock-out mice in the second phase of the formalin test as well as in mechanical allodynia during the zymosan model. Interestingly, Snca knock-out mice showed a reduced acute nociceptive response in the first phase of the formalin test which, however, is rather due to an unexpectedly enhanced response of wild type mice possibly related to the mixed C57BL/6; 129 background. In accordance with the missing effect in the inflammatory behavioral response, there was also no difference in wild type and Snca knock-out mice concerning the regulation of the proinflammatory gene COX-2 and the neuronal activity marker c-fos.

While formalin injection induced upregulation of αSyn in the spinal cord, the SNI surgery led to a late decrease of αSyn protein in wild type mice as assessed by immunofluorescence and western blot analysis. This might be associated with a counter-regulation of the protein involved in regeneration processes. In behavioral analysis, Snca knock-out mice revealed a significantly lowered neuropathic pain behavior in the late phase of the model also.

Previous publications showed relations of αSyn with endogenous antinociceptive mechanisms. Interactions of αSyn with the delta-opioid receptor DOR were described [37,40], and it was suggested that overexpression of α-Syn leads to the down-regulation of DOR [12]. In wild type and Snca knock-out animals in our model, basal DOR levels were similar, however nerve injury induced a downregulation of DOR in the knock-out mice. Similarly, there were hints that a deletion of αSyn might increase the endocannabinoid receptors system [41] which could not be confirmed in our experiments. Therefore, antinociceptive mechanisms in the spinal cord of αSyn knock-out mice appear more inhibited than activated. On the other hand, we observed a significant downregulation of MAPK activity and inflammatory pathways in the SNI model of neuropathic pain, thus suggesting that the inhibition of pronociceptive pathways contributes to the antinociceptive effects of αSyn deletion.

So far, there are only a few reports on the role of αSyn inhibition in neuronal insults and pain, since most publications focus on the αSyn overexpression that occurs in Parkinson´s disease. However, it has already been shown in rodent models of ischemia that αSyn protein expression and nuclear translocation are upregulated in neurons of the brain and spinal cord [42,43]. Knockdown or knock-out of αSyn significantly decreased the infarction size and neuronal damage and improved motor function recovery associated with decreased mitochondrial fragmentation, oxidative stress, apoptosis, and autophagy [44]. In models of spinal cord injury (SCI), αSyn knock-out mice revealed significantly decreased neuronal damage in the injured spinal cord. On the other hand, αSyn knock-out appeared in concert with reduced locomotor recovery after SCI indicating that αSyn is important in motor coordination [45] which, however, is not affected basally by αSyn inhibition as shown in our motor coordination experiments with naïve KO mice. In addition, pro-inflammatory responses in the spinal cord were significantly inhibited in KO mice, as indicated by less macrophage accumulation and lower proinflammatory cytokine levels associated with reduced NF_K_B activation [45]. Further studies showed that overexpression of αSyn induces a pro-inflammatory phenotype in astrocytes associated with the increased release of proinflammatory mediators such as TNFα and CXCL1 [46]. It was speculated that high α-synuclein in astrocytes contributes to a further loss of spinal motor neurons [47]. The downregulation of α-synuclein ameliorated tissue damage and reduced inflammation in SCI [48].

These observations are in line with our data showing a decreased invasion of immune cells and reduced levels of the inflammatory mediators IL1β, TNFα and COX-2 levels in the spinal cord of Snca knock-out in comparison to wild type mice after nerve injury. Interestingly, in inflammatory nociceptive models, we observed no effect of αSyn inhibition on proinflammatory cytokines and proteins which, however, fitted well with the lack of effect on the nociceptive behavior in these models. This might be due to the early time point of 2 h after formalin injection which rather induces neuronal than late proinflammatory immune cell responses. In contrast, in the SNI model of neuropathic pain, upregulated proinflammatory mediators in the spinal cord might at least partially be attributed to the activation of immune cells such as microglia and astrocytes.

MAP kinases are important contributors to pain sensitization after nerve injury [49]. A potential interaction between MAP kinase signaling and αSyn has already been described, with overexpression of αSyn associated with upregulation of p38 and p42/44 activity [50,51,52]. In addition, inhibition of p38 was linked to protective effects in PD and to a decrease in microglia activation [51]. Therefore, downregulation of MAPK activation in Snca knock-out mice after nerve injury associated with decreased mechanical allodynia in our study fits very well with the already published data.

## 5. Conclusions

Taken together, our results show that α-synuclein is involved in mechanisms of neuropathic pain in an experimental model of peripheral neuropathy. The inhibition of αSyn does not enhance endogenous inhibitory proteins such as CB1 and DOR, but is associated with a reduced inflammatory response as well as suppression of pronociceptive MAP kinase signalling. Further studies are necessary to clearly identify the complete αSyn mechanisms in pain, however our results already indicate that αSyn might constitute a potential target for the treatment of neuropathic pain.

## Figures and Tables

**Table 1:** Motor function and acute nociception of Snca wild type and knock-out mice, Hanging wire and rotarod test were performed to assess motor functions, hot plate and cold plate test to analyse acute thermal nociception, and the dynamic plantar test was used for acute mechanical sensitivity. (n.s. = not significant).

Test System	Snca Wild Type (Latency Time, Mean ± SEM)	Snca Knock-Out (Latency Time, Mean ± SEM)	Significance/n
**Hanging Wire**	60 s	60 s	n.s. (*n* = 12)
**Rotarod**	90 s	90 s	n.s. (*n* = 12)
**Cold Plate**	23.93 ± 1.59 s	34.88 ± 2.11 s	*** *p* < 0.001 (*n* = 11)
**Hot Plate**	13.58 ± 0.99 s	16.32 ± 1.29 s	n.s. (*n* = 12)
**Dynamic Plantar**	9.17 ± 0.44 s	8.54 ± 0.42 s	n.s. (*n* = 12)

## Data Availability

Not applicable.

## Supplementary Materials

The following are available online at https://www.mdpi.com/article/10.3390/cells11121967/s1, Figure S1: Expression, regulation and functions of aSyn in the DRGs; Figure S2: Zymosan induced paw inflammation; Figure S3: Mechanical and cold allodynia in the SNI model; Figure S4: Expression of delta-opioid receptor (DOR) and cannabinoid receptor 1 (CB1 R) genes in the spinal cord of mice in the SNI model; Figure S5: Immunofluorescence background control staining.
