# Supplementary material for: The Role of AlphαSynuclein in Mouse Models of Acute, Inflammatory and Neuropathic Pain
Source: Cells. 2022 Jun 19;11(12):1967. doi: 10.3390/cells11121967 (PMC9221919; doi:10.3390/cells11121967)
Supplement: Supplementary file 1 [file cells-11-01967-s001.zip › cells-1723500-supplementary.pdf]

Suppl Figure S1: Expression, regulation and functions of αSyn in the DRGs

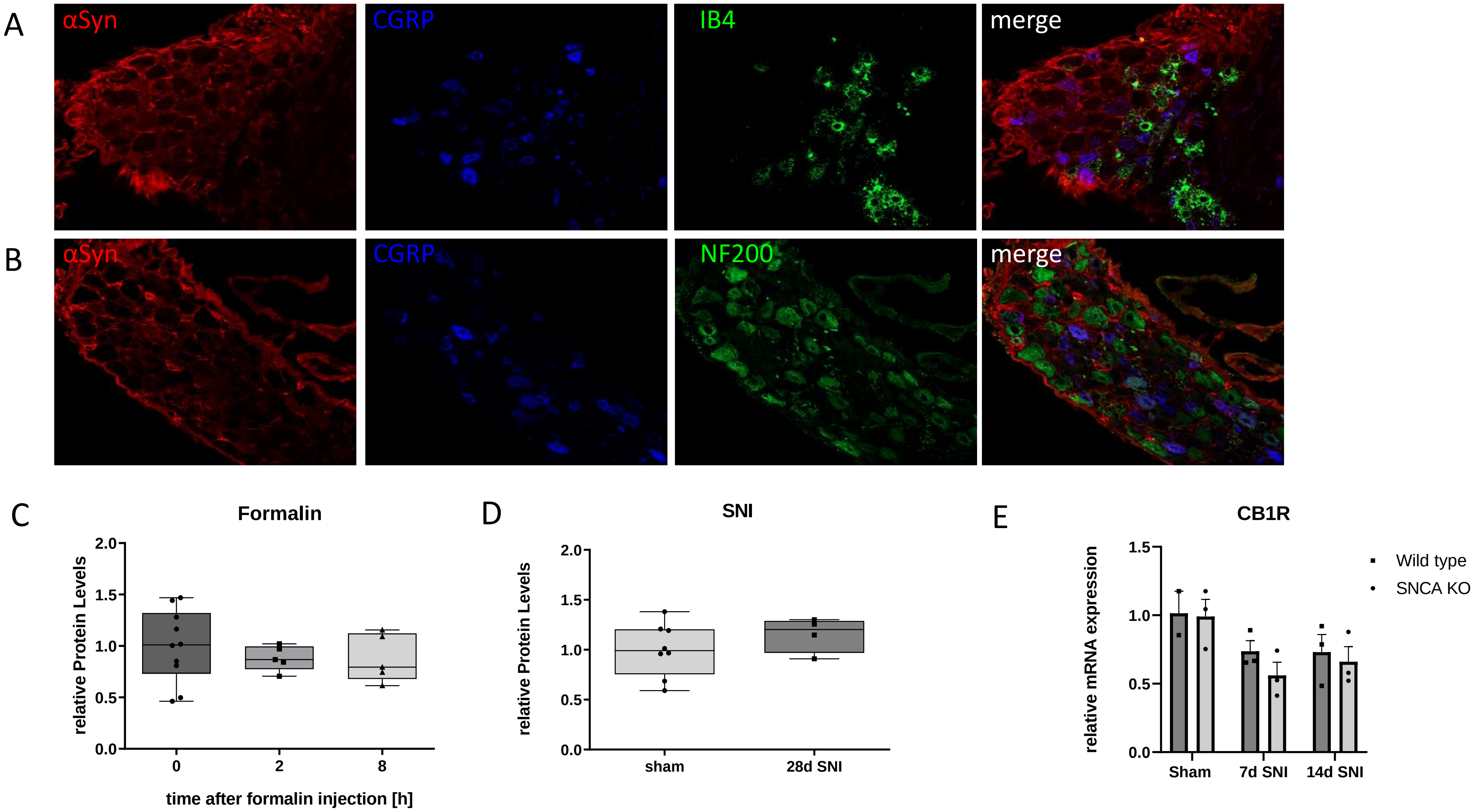

Suppl. Figure S1: Expression, regulation and functions of αSyn in the DRGs. (A) immunofluorescence showing αSyn (red), CGRP (blue), IB4 (green) and merge, (B) immunofluorescence showing αSyn (red), CGRP (blue), NF200 (green) and merge in the DRG of wild type mice, (C) Protein regulation of αSyn in the DRG after formalin injection into the paw (n = 4-10), (D) Protein regulation of αSyn in the DRG in sham and SNI-treated mice (n = 4-8), (E) Regulation of CB1R mRNA in the DRG of Wild type and Snca knock-out mice after sham and SNI treatment (n =3).

Suppl Figure S2: Comparison of wild type and Snca knock-out mice in the zymosan induced paw inflammation model

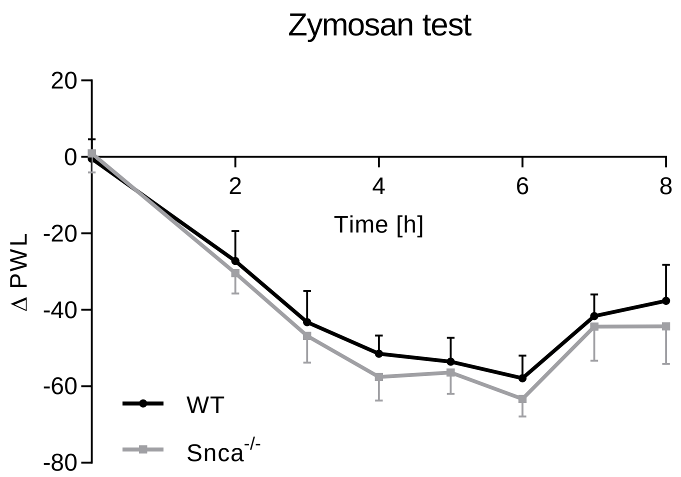

Suppl. Figure S2: Zymosan-induced paw inflammation. Dynamic plantar test to assess mechanical allodynia in the paw after injection of zymosan A in wild type and Snca knock-out mice. The diagram shows the delta paw withdrawal latency (Δ PWL) which was calculated as the percentage ratio between the ipsi- and contralateral paw. n = 7-8.

Suppl Figure S3: Mechanical and cold allodynia in the SNI model

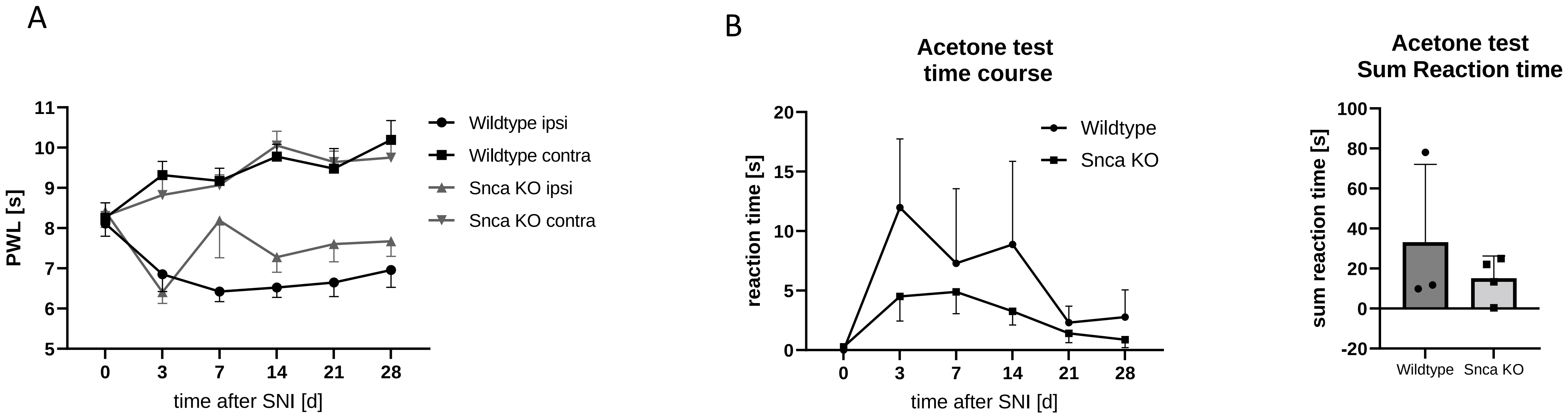

Suppl. Figure S3: Mechanical and cold allodynia in the SNI model, (A) Paw withdrawal latencies of ipsi- and contralateral paws in the SNI**.** Dynamic plantar test to assess mechanical allodynia in wild type and Snca knock-out mice after SNI. n = 12 WT/13KO. (B) Cold allodynia as assessed in the acetone test after SNI surgery. The left diagram shows the time course after SNI, the right diagram the total reaction time per group (n =3).

Suppl Figure S4: Regulation of genes in the endogenous antinociceptive system

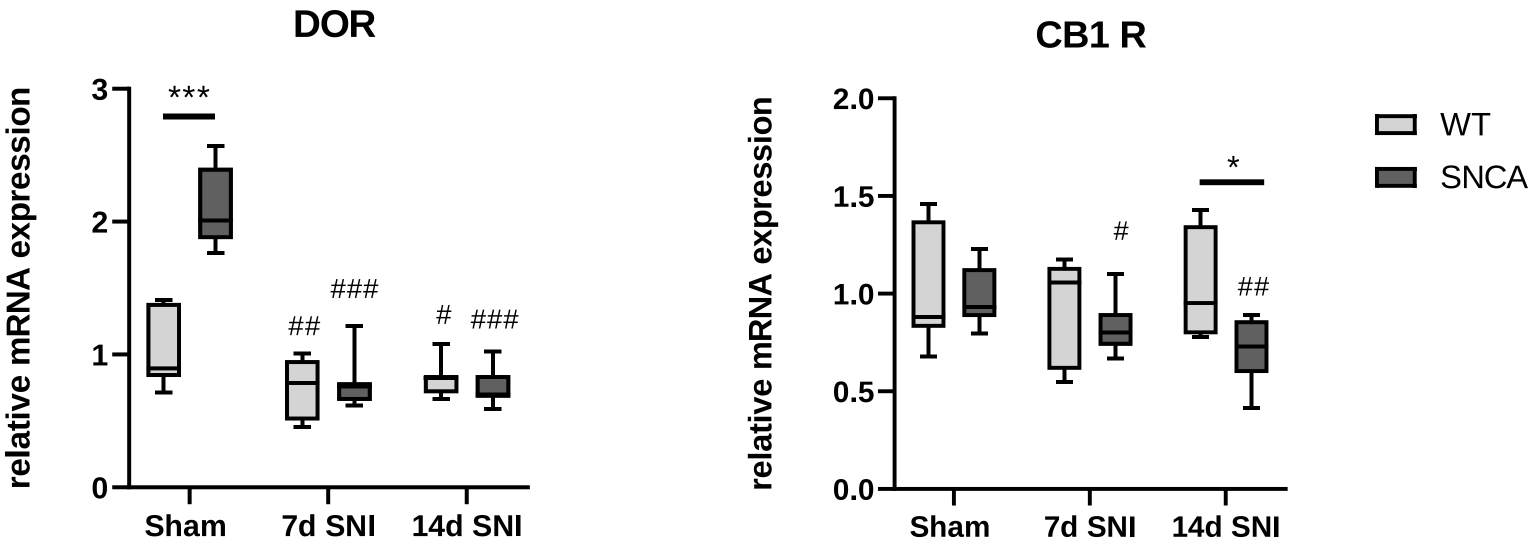

Suppl. Figure S4: Expression of delta-opioid receptor (DOR) and cannabinoid receptor 1 (CB1 R) genes in the spinal cord of mice in the SNI model. Relative mRNA expression of DOR and CB1 R in the spinal cord of wild type and SNCA knock-out mice after sham or 7 and 14d after SNI surgery, respectively. For better comparison, the wild type sham control has been set as 1. *p<0.05, *** p<0.001 statistically significant difference between the genotypes, #p<0.05, ##p<0.01, ###p<0.001 statistically significant difference between Sham and SNI.

Suppl Figure S5: Immunofluorescence background

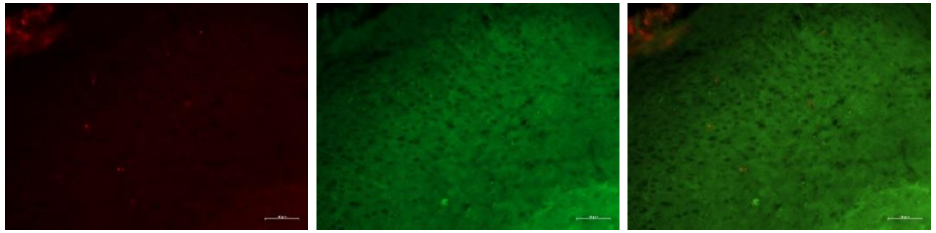

Suppl. Figure S5: Immunofluorescence background control staining, immunofluorescence was performed with the same conditions as described but without primary antibodies. Left: Cy-3 background, middle: AlexaFluor-488 staining, right: merge, Scale bars = 50 µm
